# Tea: From Historical Documents to Modern Technology

**DOI:** 10.3390/molecules28072992

**Published:** 2023-03-27

**Authors:** Liang Zhang, Yongquan Xu, Zhonghua Liu

**Affiliations:** 1 State Key Laboratory of Tea Plant Biology and Utilization, Anhui Agricultural University, Hefei 230036, China; 2 Key Laboratory of Tea Biology and Resources Utilization, Tea Research Institute Chinese Academy of Agricultural Sciences, Ministry of Agriculture and Rural Affairs, 9 South Meiling Road, Hangzhou 310008, China; 3 Key Laboratory of Tea Science of Ministry of Education, Hunan Agricultural University, Changsha 410128, China

**Keywords:** tea, processing, flavor, health benefits, mechanical processing

## Abstract

Tea is among the most important beverages globally. The spread of tea from the East to West has not only affected lifestyles, but also promoted the exchange of exchange between the East and West. Tea processing, which is critical for the development of tea flavor, includes multiple steps, such as withering, deactivation, rolling, fermentation (enzymatic oxidation) and post-fermentation. With the development of mechanical processing, tea has now become widely produced, both by hand-crafting and mechanical processing. Multiple components of tea, such as tea polyphenols, theanine, tea pigments and caffeine, have also been acquired by modern separation techniques. In traditional Chinese medicine, tea has long been documented as beneficial to health. Modern medical and nutritional studies have demonstrated that tea has many health benefits, acting to lower blood lipids, blood sugars, anti-inflammation, and anti-oxidation. To some extent, the activities of tea verified by modern medicine are consistent with the recordings in traditional medicine. Interdisciplinary theories, methods and techniques will contribute bridging knowledge contained within historical documents on tea and modern technology and science.

Recently, China’s traditional tea-making industry entered UNESCO cultural heritage list [1,2]. Tea is not only a popular beverage around the world, but also a comprehensive symbol incorporating Chinese culture and spiritual pursuits. The synonym of “茶(tea)” has existed in the history of China for about two thousand years.

Throughout history, tea had been tightly connected with detachment and spiritual enjoyment, and it is believed that tea was firs artificially planted, produced and consumed by monks. “Cha Jing (Tea Classic)”, written by Lu Yu in Tang Dynasty, was the first tea book in world history. The book describes and briefly summarizes the source, planting, picking, processing, brewing, and drinking of tea from the Western Han Dynasty to the Tang Dynasty [3].

In the 1800s, with the development of tea cultivation and processing from China to India, black tea became one of the most important agricultural trade commodities, and affected the lifestyle in Europe and America. Tea trading played a strong role the American Revolution and the First Opium War between the Qing Dynasty of China and British Empire through the butterfly effect [4,5,6].

In the 20s century, Wu Jue-Nong reviewed and expanded the content of “Cha Jing (Tea Classic)” by writing the book “Review of Tea Classic”, which began to explain the basic scientific theories of tea processing and health benefits [7].

In the modern tea industry, black tea production has been at the forefront of the development of the whole tea industry. CTC black tea, a representative industrial manufactured tea, goes beyond the traditional handmade teas in many aspects. The mechanical processing of tea and diverse utilization of tea products has not only promoted a revolution in tea production, but also prolonged the industrial chain of tea. In 2021, the total yield of tea on the Chinese mainland was about 3.18 million tons and the numbers of tea brands (products) were in the hundreds, meeting customer requirements [8].

Unlike tea products from other areas, Chinese tea products are divided into many different categories, and each category contains dozens to hundreds of tea products with different aromas, tastes, shapes, colors, etc. [9]. The basic processing steps of different teas are similar, but with minor differences in terms of parameters. These minor changes will determine the flavor characteristics of each type of tea [10]. For example, it is well known that shaking promotes the transformation of lipids into volatiles and improves the flowery perception of oolong tea, whereas withering with different time changes the aroma compositions of white tea [11,12,13,14].

In terms of the sensory attributes of tea, the color is perceived by eyes, the aroma volatiles are captured by the receptors of olfactory bulb cells, and most taste compounds bind with the human taste receptor or interact with salivary proteins to produce basic taste senses or astringency when the tea infusion is sipped [10,15,16].

We have launched this Special Issue of *Molecules,* entitled “Tea Processing and Flavor Research”, to unveil the associations between processing and flavor. Indeed, the flavor chemistry and characteristics of tea is tightly correlated to tea processing. The sensory characteristics of tea infusion are worthy of a complicated investigation into the processing, brewing and drinking. In this Special Issue, we invite authors to submit theoretical basic studies on chemical variation of tea compounds during processing, the flavor chemistry and related novel techniques, and the application of technologies the improve. Short reviews addressing tea processing and flavor also lie within the scope of this Issue.
